# The effect of the dietary approaches to stop hypertension diet on total antioxidant capacity, superoxide dismutase, catalase, and body composition in patients with non-alcoholic fatty liver disease: a randomized controlled trial

**DOI:** 10.3389/fnut.2023.1163516

**Published:** 2023-10-20

**Authors:** Zahra Zare, Mahdieh Hosseinzadeh, Fatemeh Sharuni, Fatemeh Sadat Rohani, Hashem Hojjat, Shahab Rahimpour, Farzan Madadizadeh, Javad Zavar Reza, Alexei Wong, Azadeh Nadjarzadeh

**Affiliations:** ^1^Department of Nutrition, School of Public Health, Shahid Sadoughi University of Medical Sciences, Yazd, Iran; ^2^Research Center for Food Hygiene and Safety, School of Public Health, Shahid Sadoughi University of Medical Sciences, Yazd, Iran; ^3^Department of Radiology, Faculty of Medicine, Shahid Sadoughi Hospital, Shahid Sadoughi University of Medical Sciences, Yazd, Iran; ^4^Department of Gastroentrology, Faculty of Medicine, Shahid Sadoughi Hospital, Shahid Sadoughi University of Medical Sciences, Yazd, Iran; ^5^Departments of Biostatistics and Epidemiology, Center for Healthcare Data Modeling, School of Public Health, Shahid Sadoughi University of Medical Sciences, Yazd, Iran; ^6^Department of Clinical Biochemistry, Faculty of Medicine, Shahid Sadoughi University of Medical Sciences, Yazd, Iran; ^7^Department of Health and Human Performance, Marymount University, Arlington, VA, United States

**Keywords:** DASH diet, non-alcoholic fatty liver disease, body composition, clinical trial DASH diet, clinical trial

## Abstract

**Aim:**

Non-alcoholic fatty liver disease (NAFLD) is a condition characterized by the accumulation of fat in the liver without excessive alcohol consumption. Lifestyle modifications, such as adopting a healthy diet, represent the standard treatment for NAFLD. However, the impact of the Dietary Approaches to Stop Hypertension (DASH) diet on oxidative stress biomarkers in patients with NAFLD remains unclear. Therefore, this study aimed to determine the effect of the DASH diet on total antioxidant capacity (TAC), catalase (CAT), superoxide dismutase (SOD) levels, and body composition in overweight and obese patients with NAFLD.

**Methods:**

A total of 70 overweight and obese patients aged 1870 years were randomly assigned to either the intervention (DASH diet, *n* = 35) or the control group (control diet, *n* = 35) for 12 weeks, with both groups following a calorie-restricted diet.

**Results:**

The mean age of participants was 43.1 ± 8.1 years in the DASH group and 45.1 ± 8.6 years in the control group. At the end of the study, a significant difference was observed in the mean TAC and SOD levels between the two groups (*p* = 0.02). After adjusting for potential confounding factors, such as age, sex, diabetes, smoking, physical activity, and baseline values, the DASH diet maintained its significant effects on TAC and SOD compared to the control diet (*p* = 0.03). However, there were no significant differences in CAT levels between the two groups. Moreover, a significant reduction in visceral fat (*p* = 0.01) and a marginally significant decrease in BMI (*p* = 0.06) were observed in the DASH group compared to the control group after adjusting for potential confounders.

**Conclusion:**

In conclusion, our study showed that following the DASH diet for 12 weeks in overweight and obese patients with NAFLD has beneficial effects on TAC, SOD, and visceral fat. These findings support the use of the DASH diet as a potential therapeutic intervention for the improvement of oxidative biomarkers in patients with NAFLD.

**Clinical trial registration:**

www.irct.ir/, identifier IRCT20170117032026N3.

## Introduction

Non-alcoholic fatty liver disease (NAFLD) is a condition characterized by the accumulation of lipids in the liver, without excessive alcohol consumption (1). NAFLD is strongly linked to metabolic dysfunctions, such as obesity and type 2 diabetes (2), and can be classified into two subtypes, namely, non-alcoholic fatty liver (NAFL) and non-alcoholic steatohepatitis (NASH) (3). NAFL is the milder form, while NASH and cirrhosis represent more severe forms of the disease.

The prevalence of NAFLD is reported to be between 14% and 30% of the general population in different regions of the world (4), with rates of 21.5% to 31.5% in Iran (5). Given the rising rates of obesity and diabetes worldwide, NAFLD has the potential to become a significant disease burden in future. The pathogenesis of NAFLD is closely associated with elements of the metabolic syndrome, such as obesity, hypertension, and dyslipidemia, and thus, the management of these risk factors remains the cornerstone of NAFLD treatment (6).

Recent evidence suggests that food and nutrition play a noteworthy role in the pathogenesis and management of NAFLD (7, 8). However, there are currently no definitive dietary guidelines for the management of NAFLD due to a lack of high-quality evidence-based data pertaining to metabolic outcomes. Nevertheless, based on previous studies, patients should aim for a 5–10% weight loss, reduce their intake of saturated and trans fats, fructose, and simple carbs, and switch to sources of complex carbohydrates such as whole grains and fruits (9, 10).

As nutritional epidemiology continues to expand our understanding of the impact of food and nutrition on health, it is becoming increasingly evident that adherence to a healthy diet can help reduce the risk of NAFLD through antioxidant pathways (11). NAFLD is a condition that is believed to be driven by oxidative stress, a process resulting from an imbalance between reactive oxygen species (ROS) formation and antioxidant defenses, which can be influenced by genetic and epigenetic factors (12, 13). Recent studies have suggested that the Dietary Approaches to Stop Hypertension (DASH) diet, which promotes the consumption of whole grains, fruits, and vegetables, as well as low-fat dairy and sodium restriction, may have benefits for individuals with NAFLD, including reductions in insulin resistance (IR), serum triglycerides, inflammation, and fat accumulation (14, 15). However, few studies have measured the effect of the DASH diet on oxidative biomarkers in individuals with NAFLD, making it a crucial area for further research. Thus, this study aimed to investigate the effect of the DASH diet on oxidative stress biomarkers levels [including total antioxidant capacity (TAC), catalase (CAT), superoxide dismutase (SOD)] and body composition in overweight and obese NAFLD patients.

## Methods and materials

### Participants

The present study enrolled individuals diagnosed with NAFLD using transient elastography (liver fibro-scan), aged between 18 and 70 years, and with a body mass index (BMI) ranging from 25 to 40 kg/m^2^. The diagnosis of non-alcoholic fatty liver disease was made by a gastroenterologist. Inclusion criteria comprised individuals who did not have any of the following conditions: pregnancy and breastfeeding, hereditary hemochromatosis, a history of jejunal bypass surgery or gastroplasty, the use of hepatotoxic drugs such as calcium channel blockers and synthetic estrogens, patients with hypothyroidism, Cushing's syndrome, kidney failure, kidney stones, cardiovascular disease, individuals with a history of hepatitis B and C, Wilson's disease, supplement use (including antioxidant and anti-inflammatory supplements such as vitamin D, vitamin E, and Omega 3), and individuals who adhered to a special diet in the last 3 months. Finally, individuals who were unwilling to comply with the recommended dietary regimen were also excluded from the study.

The present study adhered to the principles outlined in the Declaration of Helsinki (16) and obtained approval from the Research Ethics Committee of Shahid Sadoughi University of Medical Sciences (Approval number: IR.SSU.SPHREC.1400.144). Before participating in the study, all patients were fully informed of the trial's design and provided written consent. The present trial was registered on the “Iranian Registry of Clinical Trials” website (www.irct.ir/, IRCT20170117032026N3).

### Study protocol

Our research was a randomized controlled trial involving 70 patients with NAFLD, recruited from gastroenterology clinics affiliated with Shahid Sadoughi University of Medical Sciences, Yazd, Iran. The selection of patients was based on predefined inclusion criteria, and they were assigned to either the intervention (DASH diet) or control (Control diet) group using a computer-generated random allocation system. The intervention group received the DASH diet, while the control group received the standard weight loss and healthy diet. Randomization was based on a random table sequence, according to a computer-generated allocation system.

In accordance with a stratified randomization approach, the study participants underwent block randomization based on their body mass index (BMI) at a 1:1 ratio (block size of 2). BMI in the randomization process was classified into two groups (overweight and obesity). This randomization process was carried out by an independent statistician who had no involvement in the collection or input of participants' data. Subsequently, the randomization list was provided to the researcher responsible for assigning participants to their respective study groups. Both the researcher and the participants were not blinded to the study allocation. The random allocation was generated by F.M., Sh.R. facilitated enrollment, and Z.Z. and F.Sh. were responsible for assigning participants to the intervention groups. The basic characteristics of the participants, including age, sex, marital status, smoking status, consumption of food supplements and drugs, and history of diabetes and other diseases, were obtained through face-to-face interviews and questionnaire completion on the day of the patient's visit to the Laboratory at Shahid Sadoughi University of Medical Sciences in Yazd, Iran. TAC was the primary outcome and other variables (CAT, SOD, and body composition) were the secondary outcomes of the study.

### Intervention and control groups

The energy requirements of the study participants were estimated by considering their basal metabolic rate, physical activity levels, and the thermic effect of food. The Harris–Benedict equation was employed to determine the basal metabolic rate. All participants had a body mass index indicating overweight or obesity. Consequently, a calorie restriction of 500 to 700 kcal lower than each individual's calculated energy requirement was imposed. Specifically, 500 kcal for those with a BMI ranging from 25 to 31 kg/m^2^ and 700 kcal for those with a BMI greater than 31 kg/m^2^ were subtracted from their total energy requirements. Participants in both groups were asked not to change their physical activity levels throughout the study.

The DASH diet included 50–55% of energy as carbohydrates, 15–20% as protein, and 30% as total fat. It was rich in fruits, vegetables, whole grains, and low-fat dairy products and low in saturated fat, cholesterol, and refined grains. The food program was rich in potassium, calcium, magnesium, protein, and fiber and received a maximum of 2,400 mg of sodium per day. Patients were asked to remove salt from the table and add only one tablespoon of salt to their food to meet this goal.

The control group received a diet that consisted of 15–20% of energy derived from protein, 50–55% from carbohydrates, and 30% from fat, to promote weight loss and overall health. To encourage adherence to the protocol, the participants received text messages (SMS) or phone calls twice a week.

For participants with diabetes, carbohydrate intake was carefully managed based on their medication regimen, with specific distribution guidelines provided for three main meals and three snacks (17). Additionally, patients were given a substitution list to aid in food selection and encourage variety in the components of each food group.

### Physical activity assessment

The physical activity levels of participants were evaluated using the International Physical Activity Questionnaire (IPAQ), a validated instrument widely utilized in research studies (18). Metabolic Equivalent (MET) values were assigned to the recorded quantities of physical activity and subsequently categorized into three groups based on their level of intensity: very low (≤ 600 MET-min/week), low (601–3,000 MET-min/week), and moderate to high (>3,000 MET-min/week).

### Dietary intake assessment

A 3-day food record, consisting of 1 weekend day and two non-consecutive weekdays, was utilized to assess the dietary intake of participants at both the baseline and post-intervention stages. Moreover, phone interviews were employed to evaluate participants' compliance with the prescribed dietary regimen.

### Anthropometric and body composition assessment

The participants' height was measured using a stadiometer (Seca, Hamburg, Germany) while they were in a standing position with shoulders and hips against a wall and without shoes. Weight was measured using a digital scale (808Seca, Germany) while wearing light clothes and without shoes and recorded to the nearest 0.1 kg. Body mass index (BMI) was calculated by dividing weight by height squared and expressed in kg/m^2^.

The waist circumference (WC) was measured according to a standard protocol using a tape measure placed between the lower rib and iliac crest. Waist circumference (cm) was then divided by hip circumference to determine the waist-to-hip ratio (WHR) (cm). Muscle mass, body fat mass, and visceral fat percentage were evaluated using bioelectric impedance analysis (Omron KaradaScan).

### Biochemical assessments

Venous blood samples of 10 mL were obtained from all participants after a 7- to10-h fasting period. Subsequently, the samples underwent processing and serum separation before being stored at −70°C until further analysis. TAC, CAT, and SOD concentrations were determined via colorimetric assays conducted with NavandSalamat kits from Iran. The analysis was performed using an ELISA reader (Stat Fax 4200, Awareness Technology).

### Statistical analysis

The statistical analyses were performed using the Statistical Package for the Social Sciences (SPSS) version 24 software (Chicago, IL, USA). The normality of the quantitative data was assessed via the Kolmogorov–Smirnov test. Differences in qualitative variables between the two groups were compared using the chi-Square test. Intragroup changes were evaluated using the paired *t*-test, while the independent samples student *t-*test was employed to compare the average changes in the intervention group with the control group. Furthermore, to compare variables after controlling for confounders such as age, sex, diabetes status, education, baseline values, physical activity, and smoking, analysis of covariance (ANCOVA) was conducted. A *P* < 0.05 was employed to determine statistical significance. The sample size calculation for detecting a difference of 2 units of TAC between groups at a two-tailed alpha level was based on the study by Razavi Zadeh et al. (19). The calculation was performed with a 95% confidence interval and 80% power, taking into account a 2-unit change in TAC. The resulting sample size was determined to be 32 participants for each group. To account for potential drop-outs, the sample size was increased to 35 participants, representing a 10% drop-out rate (20).

## Results

### General characteristics

**Figure 1:**
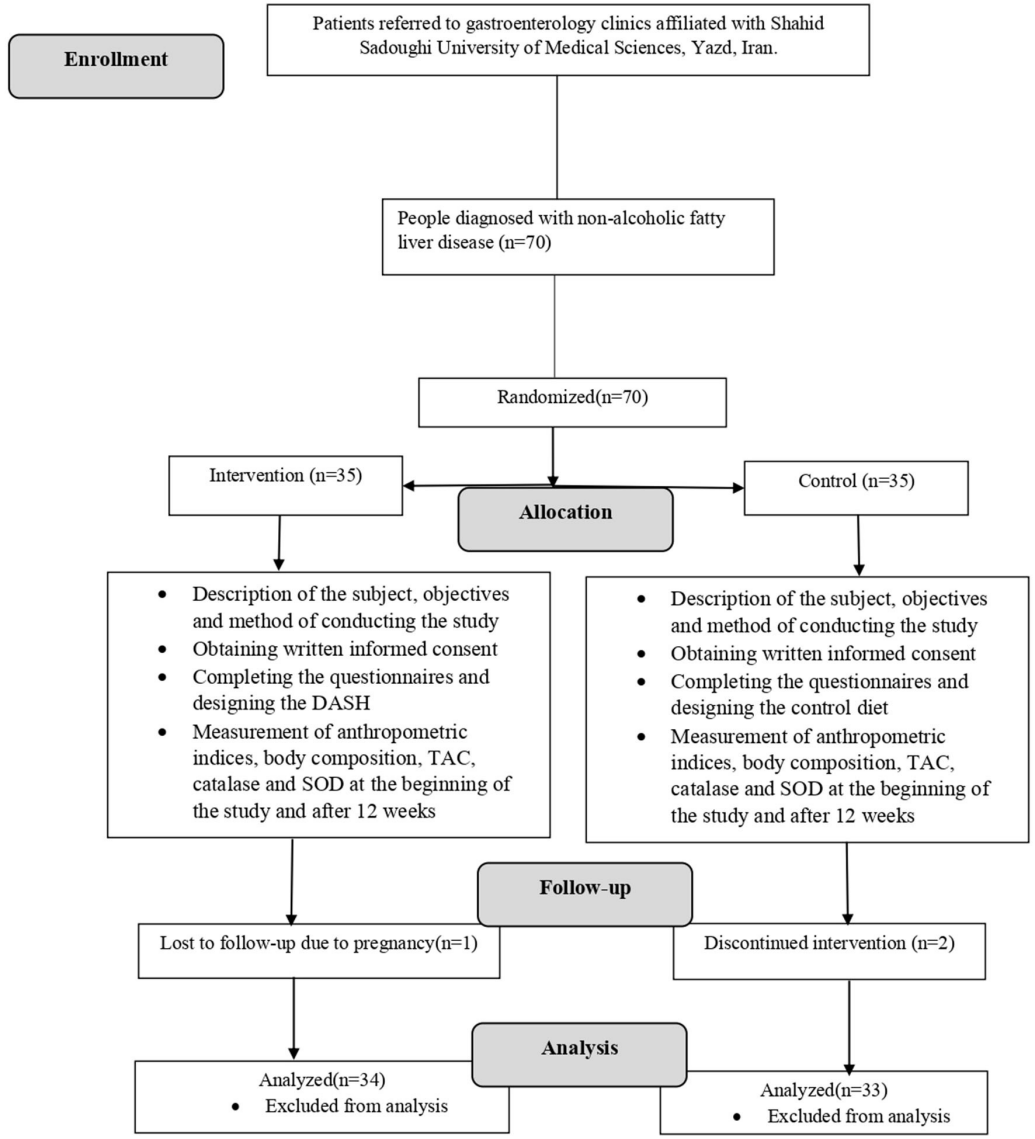
Consort flow chart of the study.

From January 2022 to April 2022, we enrolled 70 patients with NAFLD. The follow-up period was extended for 3 months beyond April. Out of the initial 70 participants, 34 individuals were assigned to the DASH diet group, while 33 were allocated to the control diet group. One participant from the DASH diet group was excluded from the study due to pregnancy, and two individuals from the control diet group were removed due to non-compliance with the dietary regimen. The baseline characteristics of the participants are shown in Table 1. Figure 1 has presented the flow chart of the study. There were no statistically significant differences between the two groups in terms of age, sex, smoking status, physical activity level, height, weight, or marital status. However, participants in the DASH diet group demonstrated higher levels of education (*p* = 0.04).

**Table 1:** Baseline characteristics of patients with NAFLD.

**Variables**		**DASH diet group (*n* = 34)**	**Control group (*n* = 33)**	***P***
Age, y		42.53 ± 9.47	47.06 ± 10.84	0.75
Gender, n (%)	Male, n (%)	20 (58.8)	13 (39.4)	0.89
	Female, n (%)	14 (41.2)	20 (60.6)	
Smoking, n (%)	No, n (%)	21 (61.8)	26 (78.8)	0.30
	Yes, n (%)	13 (38.2)	7 (21.2)	
Marital status, n (%)	Single, n (%)	8 (23.53)	6(18.18)	0.39
	Married, n (%)	26(76.47)	27(81.82)	
Education, n (%)	Under diploma, n (%)	3 (8.8)	7 (21.2)	0.034
	Diploma, n (%)	9 (26.5)	15 (45.5)	
	University, n (%)	22 (64.7)	11 (33.3)	
PA, n (%)	Low, n (%)	23 (67.65)	22 (66.66)	0.57
	Moderate, n (%)	8 (23.53)	10 (30.30)	
	High, n (%)	3 (8.82)	1 (3.04)	
Diabetes, n (%)		11(31.43)	12(36.36)	0.75
Height, Cm		167.0 ± 9.8	165.8 ± 8.7	0.65
Weight, Kg		80.3 ± 9.30	81.2 ± 12.8	0.88
FPG(mg/dl)		119.4 ± 47.0	131.1 ± 38.4	0.19

P-values are computed by independent t-test [data are expressed as mean ± standard deviation (SD)] for continuous variables and chi-square for categorical variables [data are expressed as numbers (percentage)].

NAFLD, non-alcoholic fatty liver disease; PA, physical activity; FPG, fasting blood glucose.

### Dietary intake

Table 2 shows the energy and food group intake of the participants. In comparison to the control group, participants in the DASH diet group exhibited significantly higher consumption of fruits (P < 0.001), vegetables (P < 0.001), dairy products (*p* = 0.01), and nuts, seeds, and legumes (P < 0.001), while consuming lower amounts of meat (P < 0.001) and fat (P < 0.001).

**Table 2:** Energy and food group intakes in patients with NAFLD.

**Variables**	**DASH diet group (*n* = 34)**	**Control group (*n* = 33)**	***P* ^b^**
**Energy intake, kcal/d**			
Baseline	1,996 ± 137	2,014 ± 131	0.57
After intervention	1,606 ± 315	1,527 ± 253	0.26
*P* ^a^	< 0.001	< 0.001	
*Change*			***P* ^b^**
	−389 ± 288	−478 ± 295	< 0.001
**Fruits, serving**			
Baseline	2.17 ± 0.90	2.27 ± 0.67	0.62
After intervention	4.08 ± 0.71	2.81 ± 0.63	< 0.001
*P* ^a^	< 0.001	0.001	
*Change*			*P* ^**b**^
	1.91 ± 0.99	0.54 ± 0.87	< 0.001
**Vegetables, serving**			
Baseline	1.32 ± 0.63	1.48 ± 0.75	0.34
After intervention	3.82 ± 0.57	2.81 ± 0.63	< 0.001
*P* ^a^	< 0.001	< 0.001	
*Change*			*P^***b***^*
	2.50 ± 0.92	1.33 ± 0.96	< 0.001
**Dairy products, serving**			
Baseline	1.29 ± 0.57	1.54 ± 0.50	0.06
After intervention	2.20 ± 0.41	1.84 ± 0.36	< 0.001
*P* ^a^	< 0.001	0.01	
*Change*			*P^***b***^*
	0.91 ± 0.75	0.30 ± 0.64	0.011
**Grains, serving**			
Baseline	11.7 ± 0.67	11.4 ± 0.71	0.19
After intervention	6.52 ± 1.37	7.0 ± 1.32	0.15
*P* ^a^	< 0.001	< 0.001	
*Change*			*P^***b***^*
	−5.18 ± 1.44	−4.48 ± 1.62	0.07
**Simple sugars, serving**			
Baseline	5.08 ± 0.62	5.75 ± 0.70	< 0.001
After intervention	1.02 ± 0.17	1.12 ± 0.33	0.15
*P* ^a^	< 0.001	< 0.001	
*Change*			*P^***b***^*
	−4.06 ± 0.64	−4.63 ± 0.74	0.07
**Meats, serving**			
Baseline	5.01 ± 0.73	4.78 ± 0.69	0.23
After intervention	2.79 ± 0.47	4.0 ± 0.0	< 0.001
*P* ^a^	< 0.001	< 0.001	
*Change*			< 0.001
	−2.20 ± 0.84	−0.78 ± 0.69	
**Fats, serving**			
Baseline	5.91 ± 0.66	6.12 ± 0.69	0.21
After intervention	2.76 ± 1.15	4.30 ± 0.46	< 0.001
*P* ^a^	< 0.001	< 0.001	
*Change*			*P^***b***^*
	−3.15 ± 1.26	−1.82 ± 0.85	< 0.001
**Nuts, seeds and legumes, serving**			
Baseline	0.26 ± 0.61	0.12 ± 0.33	0.24
After intervention	1.55 ± 0.56	0.60 ± 0.60	< 0.001
*P* ^a^	< 0.001	< 0.001	
*Change*			*P^***b***^*
	1.29 ± 0.72	0.48 ± 0.62	< 0.001

Values are presented as mean ± standard deviation (SD).

P^a^: The statistical analysis of the data involved comparisons within groups using a paired t-test.

P^b^: An independent t-test was employed to compare the results between the two groups.

NAFLD, non-alcoholic fatty liver disease.

### Anthropometric and body composition characteristics

Table 3 presents a comparison of pre- and post-intervention anthropometric measurements and body composition in patients with NAFLD. The mean BMI significantly decreased in both groups, with a greater reduction observed in the DASH diet group (−1.4 vs. −0.6 kg). ANCOVA analysis, adjusting for potential confounding variables such as diabetes, baseline BMI, age, sex, smoking, and physical activity, revealed a marginally significant difference in the mean change of BMI between the two groups (*p* = 0.06).

**Table 3:** Comparison of pre- and post-intervention anthropometric measurements and body composition in patients with NAFLD.

**Group**	**Baseline**	**After intervention**	**Change**	***P* ^a^**	***P* ^c^**
**BMI**					
DASH diet; *n* = 34	29.6 ± 3.4	28.2 ± 3.2	−1.4 ± 1.2	< 0.001	0.06
Control diet; *n* = 33	29.2 ± 2.6	28.6 ± 2.7	−0.6 ± 1.4	0.02	
*P* ^b^	0.54	0.72	0.02		
**WC**					
DASH diet; *n* = 34	103.5 ± 8.2	98.1 ± 8.1	−5.4 ± 4.4	< 0.001	0.14
Control diet; *n* = 33	102.6 ± 10.8	99.1 ± 9.3	−3.5 ± 6.1	< 0.001	
*P* ^b^	0.71	0.68	0.18		
**Hip**					
DASH diet; *n* = 34	108.9 ± 6.9	105.9 ± 6.7	−3.0 ± 3.6	< 0.001	0.20
Control diet; *n* = 33	107.1 ± 7.3	105.8 ± 7.9	−1.2 ± 4.0	0.11	
*P* ^b^	0.33	0.98	0.08		
**WHR**					
DASH diet; *n* = 34	0.9 ± 0	0.9 ± 0	0 ± 0	< 0.001	0.40
Control diet; *n* = 33	0.9 ± 0	0.9 ± 0	0 ± 0	0.08	
P^b^	0.79	0.61	0.67		
**VF**					
DASH diet; *n* = 34	10.7 ± 2.8	9.41 ± 2.8	−1.32 ± 0.9	0.98	0.03
Control diet; *n* = 33	10.7 ± 2.7	10.1 ± 2.5	−0.6 ± 0.9	0.32	
*P* ^b^	0.79	0.61	< 0.001		
**FFM**					
DASH diet; *n* = 34	29.9 ± 6.2	29.7 ± 5.4	−0.2 ± 2.4	0.56	0.17
Control diet; *n* = 33	29.1 ± 5.5	28.3 ± 5.2	−0.9 ± 2.0	0.29	
*P* ^b^	0.60	0.29	0.27		
**FM**					
DASH diet; *n* = 34	35.1 ± 10.1	33.4 ± 9.2	−1.7 ± 3.7	< 0.001	0.07
Control diet; *n* = 33	35.1 ± 8.8	35.5 ± 9.0	0.3 ± 4.6	0.69	
*P* ^b^	0.10	0.38	0.05		

NAFLD, non-alcoholic fatty liver disease; BMI, Body Mass Index; WC, Waist Circumference; WHR, Waist to hip ratio; VF, Visceral Fat; FFM, Fat Free Mass; FM, Fat Mass.

Values are presented as mean ± standard deviation (SD).

a The statistical analysis of the data involved comparisons within groups using a paired t-test.

b An independent t-test was employed to compare the results between the two groups.

c The mean changes between groups were compared using ANCOVA. Confounding variables, including age, gender, smoking, diabetes, education, physical activity, and baseline values, were considered during the analysis.

Paired *t*-test analysis demonstrated a significant reduction in the average WC (*p* < 0.001), WHR (*p* < 0.001), hip circumference (*p* < 0.001), and FM (*p* < 0.001) after the intervention in the DASH diet group compared to baseline values. Moreover, the ANCOVA analysis showed that after adjusting for confounders, the effect of the DASH diet compared to the control diet on visceral fat was significant (*p* = 0.03).

### Antioxidant changes

Based on the results of the independent *t*-test analysis, a significant difference was observed in the mean of change of TAC and SOD at the end of the study between the two groups (*p* = 0.02). Furthermore, after controlling for potential confounding variables, including age, sex, diabetes, baseline values, smoking, and physical activity as covariates, the DASH diet maintained its significant effects on TAC and SOD compared to the control diet.

Regarding the paired *t*-test analysis, the average SOD levels in the DASH diet group increased significantly at the end of the study compared to the baseline values (*p* < 0.001). However, in the control diet group, the increase was slight and not statistically significant (*p* = 0.87). Interestingly, no significant differences were observed within or between the groups in terms of CAT levels in both the crude and adjusted models (Table 4).

**Table 4:** Comparison of pre- and post-intervention on TAC, SOD, and CAT levels in patients with NAFLD.

**Group**	**Baseline**	**After intervention**	**Change**	***P* ^a^**	***P* ^c^**
**TAC level**					
DASH diet; *n* = 34	0.6 ± 0.2	0.7 ± 0.3	0.1 ± 0.3	0.04	0.04
Control diet; *n* = 33	0.5 ± 0.1	0.5 ± 0.2	0 ± 0.1	0.34	
*p*-value^b^	0.49	0.01	0.02		
**SOD level**					
DASH diet; *n* = 34	262.8 ± 36.3	282.7 ± 32.0	19.8 ± 26.7	< 0.001	0.02
Control diet; *n* = 33	277.9 ± 31.0	278.9 ± 42.7	1.0 ± 33.4	0.87	
*p*-value^b^	0.09	0.69	0.02		
**CAT level**					
DASH diet; *n* = 34	14.1 ± 6.0	12.1 ± 4.6	−1.9 ± 8.2	0.27	0.89
Control diet; *n* = 33	14.0 ± 7.9	11.9 ± 4.6	−2.0 ± 7.9	0.12	
*p*-value^b^	0.95	0.08	0.97		

NAFLD, non-alcoholic fatty liver disease; TAC, Total antioxidant capacity; SOD, Superoxide dismutase; CAT, Catalase.

Values are presented as mean ± standard deviation (SD).

a The statistical analysis of the data involved comparisons within groups using a paired t-test.

b An independent t-test was employed to compare the results between the two groups.

c The mean changes between groups were compared using ANCOVA. Confounding variables, including age, gender, smoking, diabetes, education, physical activity, and baseline values, were considered during the analysis.

## Discussion

In the present study, we investigated the effects of the DASH diet on oxidative stress biomarkers and body composition in overweight and obese patients with NAFLD. We found that adherence to the DASH diet for 12 weeks had significant positive effects on TAC, SOD, and visceral fat. These results remained significant even after controlling for potential confounders. While there was a marginal effect on BMI and fat mass, no significant changes were observed in CAT enzyme levels, WC, WHR, or lean mass.

Studies investigating the effects of the DASH diet on TAC, SOD, CAT, and body composition in NAFLD are scarce. Our study is consistent with previous research by Asemi et al. who found that the DASH diet intervention led to a significant increase in plasma TAC and glutathione (GSH) levels in patients with gestational diabetes mellitus (21) and obese women with polycystic ovary syndrome (22). However, Razavi Zade et al. did not find significant effects on TAC levels after 8 weeks of DASH diet consumption in overweight and obese patients with NAFLD (19).

Additionally, a recent meta-analysis of three randomized clinical trials found no significant effect of the DASH diet on TAC (23). However, it should be acknowledged that the limited number of studies included in this meta-analysis (three studies) may hinder the generalizability of this finding. The possibility that a statistically significant effect of the DASH diet on TAC exists cannot be ruled out, and more studies with larger sample sizes are needed to confirm this.

In contrast, Sureda et al. found a positive correlation between the Mediterranean diet (MD) and SOD and CAT levels in an observational study that explored associations between the MD and oxidative stress biomarkers. However, there was no effect on CAT enzyme levels in this study (24). The MD is characterized by high consumption of fruits, vegetables, grains, legumes, nuts, and seeds; low-to-moderate consumption of dairy products, fish, poultry, and wine; low intakes of red meat and eggs; and the primary source of fat utilized in the MD is olive oil (25). The common components of plant-based foods with antioxidants in both the MD and DASH diet may explain the increase in SOD levels in our study and the lack of effect on CAT enzyme levels.

Previous studies have shown that high-sugar diets may result in mitochondrial dysfunction in adipose tissue, leading to metabolic disorders and elevated levels of serum triglycerides and VLDL cholesterol, and ultimately oxidative stress (26). The DASH dietary pattern, characterized by a high intake of calcium and magnesium, has been associated with improved metabolic profiles, potentially due to the stimulation of microsomal triglyceride transfer protein (MTP) in the liver (27). This pattern may also reduce lipid peroxidation, suppress endothelial damage, and enhance antioxidant capacity in both serum and tissue (28), likely due to its high content of fruits and vegetables, which contain antioxidants such as alpha-carotene, beta-carotene, lycopene, and ascorbic acid (29, 30).

Furthermore, the DASH diet's high content of vitamin C, calcium, magnesium, and arginine (found in foods such as fish, soybeans, beans, lentils, whole grains, nuts, parsley, and fresh basil) may reduce oxidative stress by decreasing the activity of nicotinamide adenine dinucleotide phosphate (NADPH) oxidase, the main enzyme responsible for generating superoxide (31). The DASH dietary pattern may also restore the activity of antioxidant enzymes, eliminate oxygen radicals, and reduce serum levels of angiotensin II (31–33). Uncontrolled ROS production can lead to oxidative stress and ultimately hypertension. Therefore, oxidative stress biomarkers have a positive association with hypertension and a negative relationship with plasma antioxidant activity, as reflected by the activity of enzymes such as SOD and CAT (34). Since the DASH diet has been proven to have a beneficial effect on hypertension, it may increase plasma antioxidant activity by reducing ROS production. Additionally, reducing the high consumption of saturated fatty acids and non-esterified fatty acids by following the DASH diet may deactivate pro-inflammatory pathways and reduce oxidative stress, ultimately improving endothelial function (35). However, further studies are required to fully elucidate the mechanisms through which the DASH diet affects metabolic and oxidative health.

The results of our study did not show significant effects on catalase enzyme activity. There could be several possible explanations for this outcome. One possibility is that the concentration of hydrogen peroxide (H_2_O_2_) used in our experiment was not high enough to induce an increase in catalase activity. Additionally, it is possible that other enzymes that consume H_2_O_2_, such as glutathione peroxidase, may have increased and offset any potential increase in catalase activity. Unfortunately, we did not measure the activity of these other enzymes in our experiment. In contrast, previous research has shown that a high-fat diet can significantly increase catalase activity. However, we can justify our findings by pointing out that the DASH diet, which was used in our study is not high in fat and may therefore not lead to a significant increase in catalase activity (36).

One significant finding of our study was the effect of the DASH diet on visceral fat, which was significant, as well as a marginal effect on BMI and FM, but no significant effect on FFM, WC, hip circumference, and WHR. These results are consistent with previous studies that have shown the potential benefits of the DASH diet in reducing abdominal fat and visceral fat accumulation.

For instance, a study by Navarro-Prado et al. (37) involving 244 healthy young adults found that following the DASH diet was associated with a reduction in blood pressure, visceral fat, and WC. It is widely accepted that visceral fat cells can have harmful effects on the liver due to the release of inflammatory cytokines and adipokines compared to surrounding tissues (38). However, the consumption of fruits, vegetables, and legumes, along with lower saturated fatty acid (SFA) and higher monounsaturated fatty acid (MUFA), may explain the beneficial effects of the DASH diet on reducing abdominal and visceral fat accumulation (37). These findings are also in line with the research by Razavi Zade et al., who found that 8 weeks of the DASH diet led to significant declines in BMI in adults with NAFLD (19). Moreover, Asami et al. demonstrated that the DASH diet with calorie restriction led to a significant reduction in BMI in obese and overweight women with polycystic ovary syndrome (22).

While WC decreased significantly in both the DASH diet and control groups in our study, it was greater in the DASH diet group; although this difference was not significant compared to the control group. These results are in line with the study by Yazici et al. who reported a significant reduction in WC in pre-hypertensive subjects following the DASH dietary pattern for 20 weeks (39). Moreover, our findings were consistent with the study by Hasanian-Fard et al. which showed that the DASH diet combined with exercise led to a significant decrease in WHR in NAFLD patients (40). Furthermore, Razavi Zade et al., observed a greater reduction in waist and hip circumferences in the DASH group (19).

The reduction of four anthropometric indices (BMI, WC, hip circumference, and WHR) in both DASH and control groups is likely related to energy restriction (41). The family of insulin-like growth factors (IGFs) and their binding proteins appear to be involved in this process as they are differentially affected by energy restriction and weight loss (42). Short-term energy restriction leads to a decrease in insulin concentration, which increases IGF-binding protein-1 (42–44). This increase stimulates lipolysis and causes more free fatty acids to be released into the bloodstream, which can lead to the accumulation of triglycerides in the liver. Therefore, weight loss may exacerbate fatty liver disease by increasing the accumulation of triglycerides in the liver.

In the DASH group, there was a significant decrease in FM compared to the control group, but there were no significant changes in FFM. These findings are consistent with Mahdavi et al.'s study on body composition, which showed a significant decrease in FM but not FFM in hemophilic adolescents receiving the DASH diet (45). The most effective factor in reducing total body weight is the reduction of body fat, which can improve overall health status. However, there is no specific cutoff level for body fat that defines obesity in men and women, making it difficult to relate these results to health outcomes (46). Based on the results of this study in people, the DASH diet may be a potential way to improve body composition, which could lead to improved obesity management and NAFLD disease outcomes.

The strength of our investigation includes its randomized clinical trial design, which is recognized for its rigorous methodology in clinical research, enhancing the credibility of our findings. Moreover, to the best of our knowledge, this investigation represents the pioneering effort to examine the effect of the DASH diet on oxidative stress factors (TAC, SOD, and CAT) among patients with NAFLD. Additionally, accurate assessment of non-alcoholic fatty liver was achieved using the fibro-scan method, ensuring precise measurement of participants' liver condition. Both male and female participants were included in the study, contributing to a more comprehensive understanding of the diet's effects across genders.

Despite the strengths of our investigation, some limitations should be considered. Our sample consisted of overweight and obese individuals with NAFLD, and therefore, the generalizability of our findings to other populations may be limited. Additionally, although we utilized a validated questionnaire, physical activity and food intake were self-reported which may introduce bias (47). It is challenging to discern whether the beneficial effects of the DASH diet are solely attributed to increased intake of fruits and vegetables or other dietary variables. The absence of serum vitamin C level measurements (a reliable biomarker indicative of participant adherence to the DASH diet) stands as another limitation. Finally, the absence of blinding in the study design introduces the potential for bias, although this limitation should be weighed in the context of the broader study design and execution.

## Conclusion

In conclusion, our study suggests that the DASH diet may be a promising dietary option for individuals with NAFLD as it has the potential to reduce BMI, visceral fat, and antioxidant indices. However, further studies are necessary to explore other potential benefits of the DASH diet for this population.

## Data availability statement

The raw data supporting the conclusions of this article will be made available by the authors, without undue reservation.

## Ethics statement

The present study adhered to the principles outlined in the Declaration of Helsinki (16) and obtained approval from the Research Ethics Committee of Shahid Sadoughi University of Medical Sciences (Approval number: IR.SSU.SPHREC.1400.144). Prior to participating in the study, all patients were fully informed of the trial design and provided written consent. The studies were conducted in accordance with the local legislation and institutional requirements. The participants provided their written informed consent to participate in this study. Written informed consent was obtained from the individual(s) for the publication of any potentially identifiable images or data included in this article.

## Author contributions

ZZ, AN, and MH contributed to the conception and design of the study. ZZ, FS, FR, HH, SR, and JZ participated in data acquisition. ZZ, AN, and FM contributed to data analysis and data interpretation. ZZ and AN participated in manuscript drafting. AN finalized the manuscript. All authors contributed to the article and approved the submitted version.
