# Supplementary material for: The effect of the dietary approaches to stop hypertension diet on total antioxidant capacity, superoxide dismutase, catalase, and body composition in patients with non-alcoholic fatty liver disease: a randomized controlled trial
Source: Front Nutr. 2023 Oct 20;10:1163516. doi: 10.3389/fnut.2023.1163516 (PMC10624175; doi:10.3389/fnut.2023.1163516)
Supplement: Supplementary file 1 [file Table_1.docx]

| Macronutrients and micronutrients intakes in patients with NAFLD | | | |
| --- | --- | --- | --- |
| **Variables** | **Intervention group (n = 34)** | **Control group (n = 33)** | ***P*^b^** |
| **Protein**, g/d | | | |
| Baseline | 66.60 ± 12.62 | 78.18 ± 8.96 | < 0.001 |
| After intervention | 70.14 ± 13.80 | 69.93 ± 10.81 | 0.94 |
| ***P***^a^ | 0.22 | 0.001 |  |
| **Carbohydrate**, g/d | | | |
| Baseline | 254.89 ± 23.19 | 238.78 ± 24.25 | 0.007 |
| After intervention | 209.97 ± 51.08 | 197.30 ± 53.28 | 0.32 |
| ***P***^a^ | < 0.001 | < 0.001 |  |
| **Fat**, g/d | | | |
| Baseline | 78.91 ± 17.42 | 83.80 ± 13.40 | 0.20 |
| After intervention | 53.34 ± 30.59 | 51.09 ± 15.43 | 0.70 |
| ***P***^a^ | < 0.001 | < 0.001 |  |
| **Fiber**, g/d | | | |
| Baseline | 16.05 ± 6.24 | 15.07 ± 3.80 | 0.44 |
| After intervention | 33.06 ± 9.09 | 22.26 ± 9.50 | < 0.001 |
| ***P***^a^ | < 0.001 | < 0.001 |  |
| **Vitamin A**, ug/d | | | |
| Baseline | 300.47 ± 69.54 | 330.88 ± 70.36 | 0.08 |
| After intervention | 540.89 ± 60.16 | 481.54 ± 53.89 | < 0.001 |
| ***P***^a^ | < 0.001 | < 0.001 |  |
| **Vitamin C**, mg/d | | | |
| Baseline | 46.16 ± 5.59 | 46.98 ± 4.11 | 0.49 |
| After intervention | 65.84 ± 5.91 | 54.49 ± 6.19 | < 0.001 |
| ***P***^a^ | < 0.001 | < 0.001 |  |
| **Vitamin K**, ug/d | | | |
| Baseline | 41.17 ± 10.72 | 44.74 ± 12.36 | 0.21 |
| After intervention | 80.93 ± 12.56 | 62.94 ± 13.01 | < 0.001 |
| ***P***^a^ | < 0.001 | < 0.001 |  |
| **Folate**, ug/d | | | |
| Baseline | 194.33 ± 43.12 | 188.33 ± 29.15 | 0.50 |
| After intervention | 358.12 ± 66.82 | 282.87 ± 70.99 | < 0.001 |
| ***P***^a^ | < 0.001 | < 0.001 |  |
| **Sodium**, mg/d | | | |
| Baseline | 2957.66 ± 440.74 | 2854.20 ± 274.06 | 0.25 |
| After intervention | 1701.36 ± 556.10 | 1791.73 ± 606.51 | 0.52 |
| ***P***^a^ | < 0.001 | < 0.001 |  |
| **Potassium**, mg/d | | | |
| Baseline | 1922.71 ± 407.28 | 1994.11 ± 337.16 | 0.43 |
| After intervention | 3498.80 ± 667.24 | 2723.24 ± 655.55 | < 0.001 |
| ***P***^a^ | < 0.001 | < 0.001 |  |
| **Magnesium**, mg/d | | | |
| Baseline | 300.42 ± 65.73 | 310.91 ± 32.96 | 0.41 |
| After intervention | 422.42 ± 87.09 | 353.99 ± 86.13 | 0.002 |
| ***P***^a^ | < 0.001 | 0.008 |  |
| **Calcium**, mg/d | | | |
| Baseline | 705.27 ± 191.54 | 761.99 ± 157.29 | 0.19 |
| After intervention | 1116.16 ± 184.68 | 919.70 ± 169.80 | < 0.001 |
| ***P***^a^ | < 0.001 | < 0.001 |  |
| **Zinc**, mg/d | | | |
| Baseline | 11.95 ± 1.58 | 11.72 ± 1.56 | 0.54 |
| After intervention | 12.43 ± 2.21 | 12.99 ± 1.62 | 0.24 |
| ***P***^a^ | 0.23 | 0.002 |  |
| Values are presented as mean ± standard deviation (SD).  *P*^a^: resulted from comparisons within groups by paired t-test.  *P*^b^: resulted from comparisons between two groups by independent t-test.  NAFLD: non-alcoholic fatty liver disease. | | | |
